# Crowdsourced Community Support Resources Among Patients Discharged From the Emergency Department During the COVID-19 Pandemic: Pilot Feasibility Study

**DOI:** 10.2196/31909

**Published:** 2022-02-23

**Authors:** Anish K Agarwal, Lauren Southwick, Rachelle Schneider, Arthur Pelullo, Robin Ortiz, Elissa V Klinger, Rachel E Gonzales, Roy Rosin, Raina M Merchant

**Affiliations:** 1 Department of Emergency Medicine University of Pennsylvania Philadelphia, PA United States; 2 Departments of Pediatrics and Population Health NYU Grossman School of Medicine Institute for Excellence in Health Equity New York, NY United States; 3 Center for Health Care Innovation Penn Medicine Philadelphia, PA United States

**Keywords:** COVID-19, mHealth, CHW, digital health, platform, crowdsource, support, community, health system, monitoring, virtual care, text message, model, community health worker, pilot study, feasibility

## Abstract

**Background:**

The COVID-19 pandemic has placed strains on communities. During this public health crisis, health systems have created remote methods of monitoring symptom progression and delivering care virtually.

**Objective:**

Using an SMS text message-based system, we sought to build and test a remote model to explore community needs, connect individuals to curated resources, and facilitate community health worker intervention when needed during the pandemic. The primary aims of this pilot study were to establish the feasibility (ie, engagement with the text line) and acceptability (ie, participant ratings of resources and service) of delivering automated well-being resources via smartphone technology.

**Methods:**

Eligible patients (aged 18 years or older, having a cell phone with SMS text messaging capability, and recently visited the emergency department) were identified using the electronic health record. The patients were consented to enroll and begin receiving COVID-19–related information and links to community resources. We collected open-ended and close-ended resource and mood ratings. We calculated the frequencies and conducted a thematic review of the open-ended responses.

**Results:**

In 7 weeks, 356 participants were enrolled; 13,917 messages were exchanged including 333 resource ratings (mean 4) and 673 well-being scores (mean 6.8). We received and coded 386 open-ended responses, most of which elaborated upon their self-reported mood score (29%). Overall, 77% (n=274) of our participants rated the platform as a service they would highly recommend to a family member or friend.

**Conclusions:**

This approach is designed to broaden the reach of health systems, tailor to community needs in real time, and connect at-risk individuals with robust community health support.

## Introduction

### Background

The COVID-19 pandemic continues to disrupt the health and well-being of communities. An immediate call to address clinical issues has been followed by a call to public health and community action [1]. Prior to COVID-19, underserved and vulnerable communities were struggling with basic “life needs,” and a high disease burden (which led to public health actions needed to curb the epidemic) placed further strain on access tied to community resources [2]. Despite new concerns such as emerging variants [3] and reaching herd immunity to prevent wider spread in the community [4], all 50 states have reopened public spaces, businesses, and the larger economy. Although public health policies in place, such as social distancing and self-quarantines, are meant to curb the epidemic, they also can lead to social isolation and loneliness. [5]. Prior to COVID-19, communities and individuals were already struggling with loneliness and well-being, which have been shown to negatively impact physical and mental health [6].

Health care institutions are woven into the fabric of communities and serve as a critical hub of information, resources, and care. These systems will continue to play an integral part in supporting communities throughout the prolonged response to, and recovery from, the pandemic [7]. However, community members themselves are likely best equipped to identify areas of focus and what nonclinical needs should be addressed. The pandemic impacted not only the health of individuals but all aspects of society, including the climate, workplaces, and education, as well as global, national, and local economies [8].

An emerging and critical health care professional is the community health worker (CHW). The American Public Health Association defines CHWs as “frontline public health worker who is a trusted member of and/or has an unusually close understanding of the community served” [9]. The CHW has a trusting relationship with community members, which can improve access to health care and other social services [9]. The Centers for Disease Control and Prevention reported that the use of CHWS for home-based care during the COVID-19 pandemic can alleviate overburdened health care institutions. In parallel, the use of technology to adapt health care has rapidly changed during the COVID-19 pandemic [10]. Clinical providers shifted to virtual visits and text messaging-based communication to care for patients and limit physical interactions [11]. Virtual support networks have been created to rapidly shift care, and health systems remain uniquely positioned to also serve as stewards of health information and mental health or well-being support for broader communities. Emotional disaster models highlight the need for persistent vigilance and monitoring to ensure that well-being is prioritized, monitored, and proactively supported [12]. As many health systems quickly implemented clinical remote monitoring, contact tracing mechanisms, and testing and vaccination sites [13], the challenge remains in developing a scalable, remote approach toward engaging at-risk groups, identifying needs and providing tailored community mental health and well-being support.

### Objective

This pilot study’s purpose was two-fold. First, to identify patients with an initial health system encounter (emergency department [ED] discharge) early in the pandemic (April-June 2020) and engage them via SMS text messaging to understand collective community and well-being needs. Second, to curate a community-driven repository of community resources and connect individuals experiencing emotional distress or those who express other social support needs to CHWs for urgent nonclinical needs.

### Hypotheses

We hypothesize that we will be able to connect high-risk individuals to CHWs for urgent social support and nonclinical needs. Second, we will be able to collect community-identified resources and better understand collective community and well-being needs.

## Methods

### Recruitment

Our team deployed a text message-based script, and using an automated text messaging platform, the patients were consented to enroll and begin receiving COVID-19–related information and links to community resources (eg, social services, resources for families with children, mental health and well-being, and virtual, hyperlocal cultural events). The text messaging script sought to engage participants and elicit feedback on all resource content in order to prioritize the content driven by community members [14]. This study employed an iterative design approach to test assumptions and gather continuous descriptive feedback. Further details regarding the pilot design have been reported previously [15].

The participants were identified using the electronic health record (EHR) and sent an initial message if they met the following inclusion criteria: adult (>18 years of age); mobile number in EHR; and recent ED discharge. Enrollment occurred between April through May 2020. We defined this study sample as high-risk due to their ED use during the COVID-19 pandemic in Spring 2020. The participants were recruited through a Health Insurance Portability and Accountability Act (HIPAA)-compliant technology platform (Mosio), to invite eligible participants and engage in 2-way text messaging. The participants received a text message about the pilot and were asked if they wanted to opt in with a simple “yes” reply. Potential participants who did not respond or replied “stop” were no longer contacted. The entire process of approaching, enrolling, and engaging participants was completed through text messaging.

#### Measures

The participants were asked to provide quantitative ratings on resource links, mood ratings, and open-ended narrative feedback. Participants were asked to provide quantitative ratings (1-5 scale, 5=very helpful) on resource links, mood ratings, and open-ended narrative feedback weekly. Participant feedback and ratings of the resources guided the content of the mobile web-based resource hub. This allowed the content to be curated and defined by community members. This created a mechanism to continuously tailor content and create a living, community-driven resource hub.

**Figure 1:**
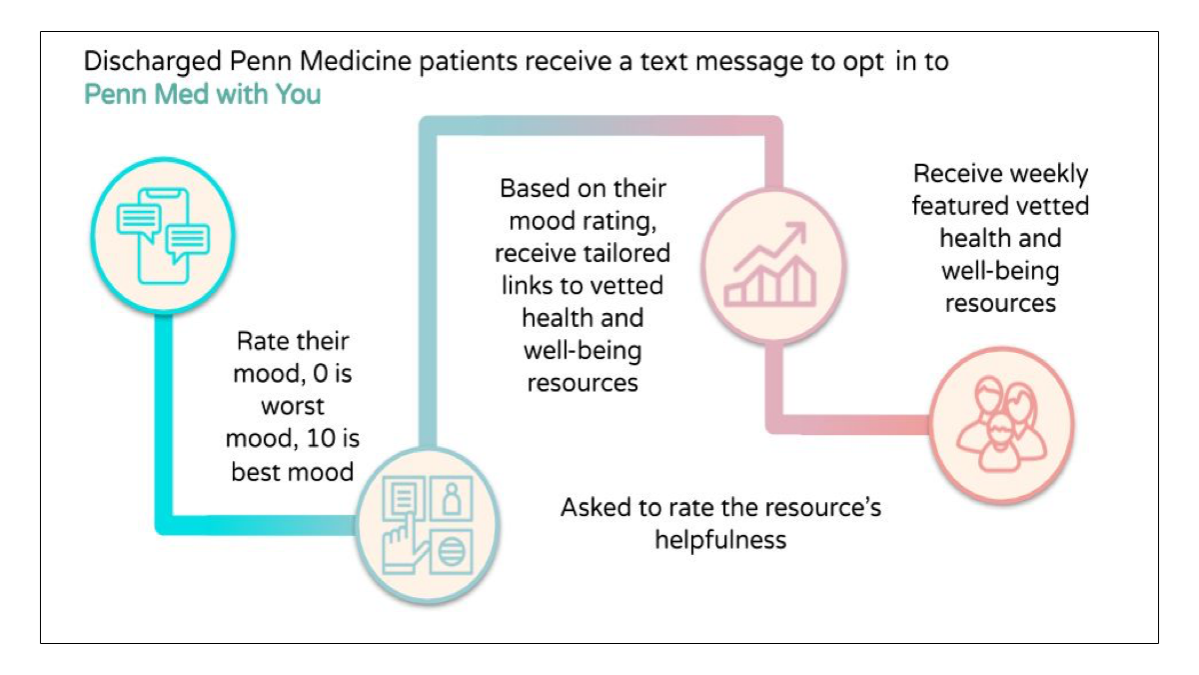
Penn Med with You participant flow.

The participants self-reported well-being on a weekly basis on a numeric scale (0-10, with 0 representing the worst mood and 10 as the best mood) [16]. A “high” mood rating was assigned for ratings between 7 and 10, “medium” mood rating between 4 and 6, and “low” mood between 0 and 3. The participants who reported a low mood (0-3) were sent a follow-up message to assess their mood compared to the previous day, “Good morning, I wanted to see how you’re doing compared to yesterday. How is your mood today? Please text back a number between 0 and 10, 0 is the worst mood and 10 is the best mood.” If the participants reported consistent low well-being scores (up to 3 days) or responded with an urgent social support need (eg, mental health issue and accessing services) they were contacted by our team and connected to a trained CHW (Figure 1). Moreover, there were open-ended and closed-ended acceptance measures such as how to improve the text line and the Net Promote Score (NPS) [17], a commonly used single-survey question about an individual’s willingness to recommend a product or service. NPS was asked midway (2-week mark) and at the end of the pilot study (4-week mark). The NPS asks, “on a scale of 1-10, how likely would you recommend this text message service to a friend? 1 is very unlikely to recommend and 10 is very likely.” NPS is scored by segmenting the respondents into 3 groups: promoters (scored 9 or 10), passives (scored 7 or 8), and detractors (scored 6 or below) and subtracting the proportion of detractor respondents from promoters. Historically, NPS scores are used in business as a predictor of growth in sales and revenue [17].

#### Procedures and SMS Text Messaging System

**Figure 2:**
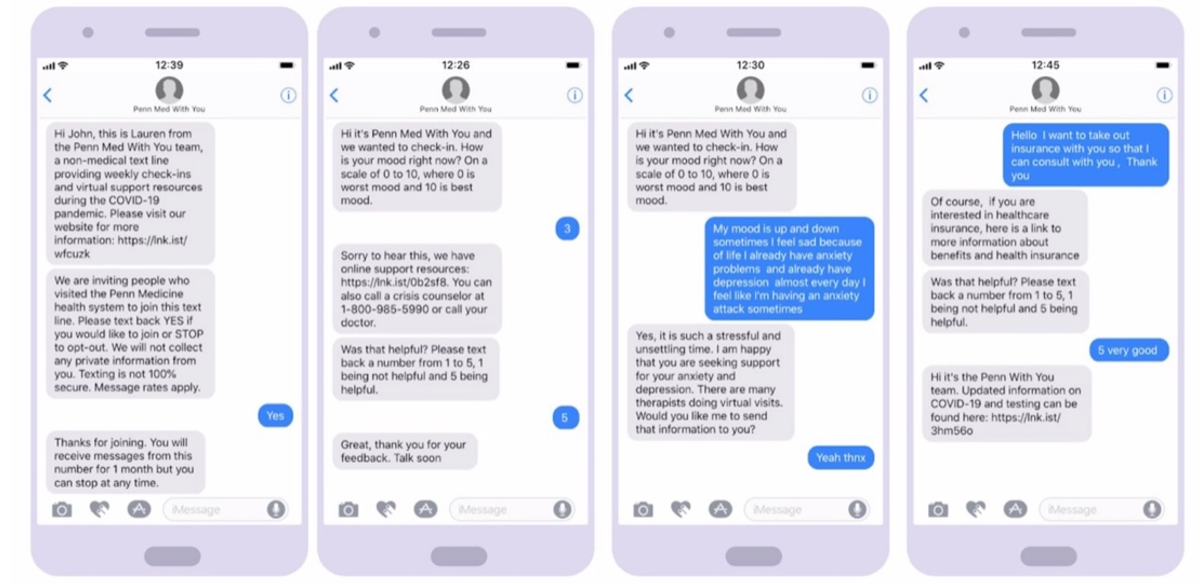
Automated text messaging engagement.

**Figure 3:**
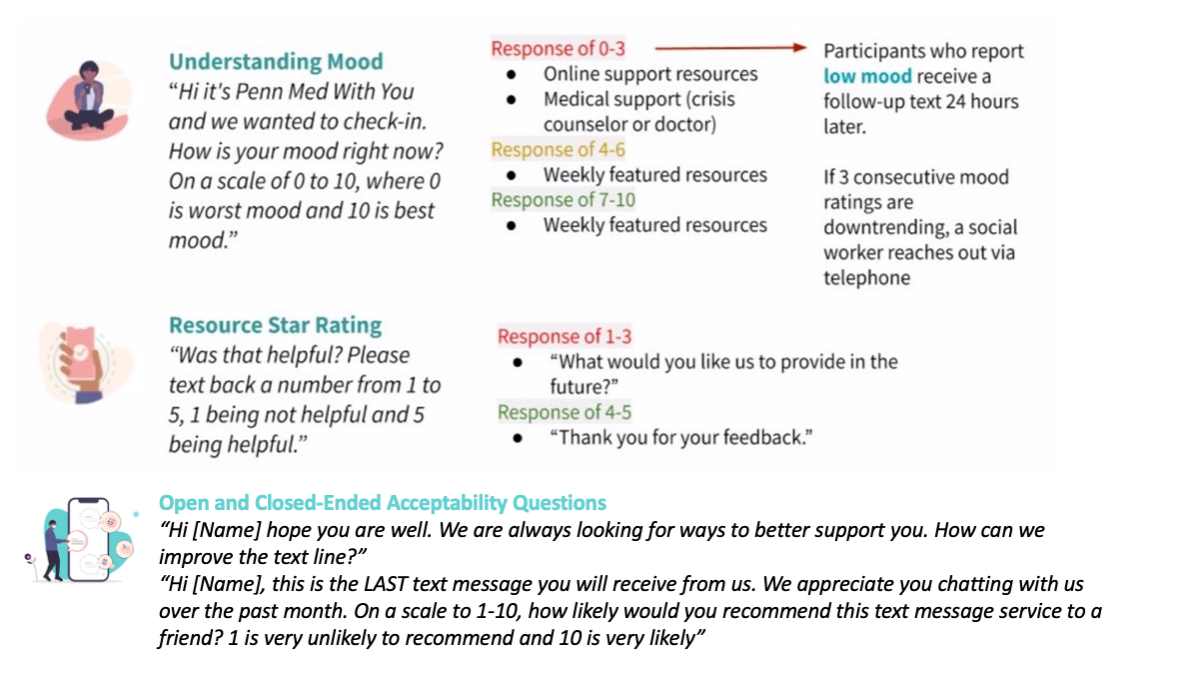
Tailored messaging schematic for well-being.

**Figure 4:**
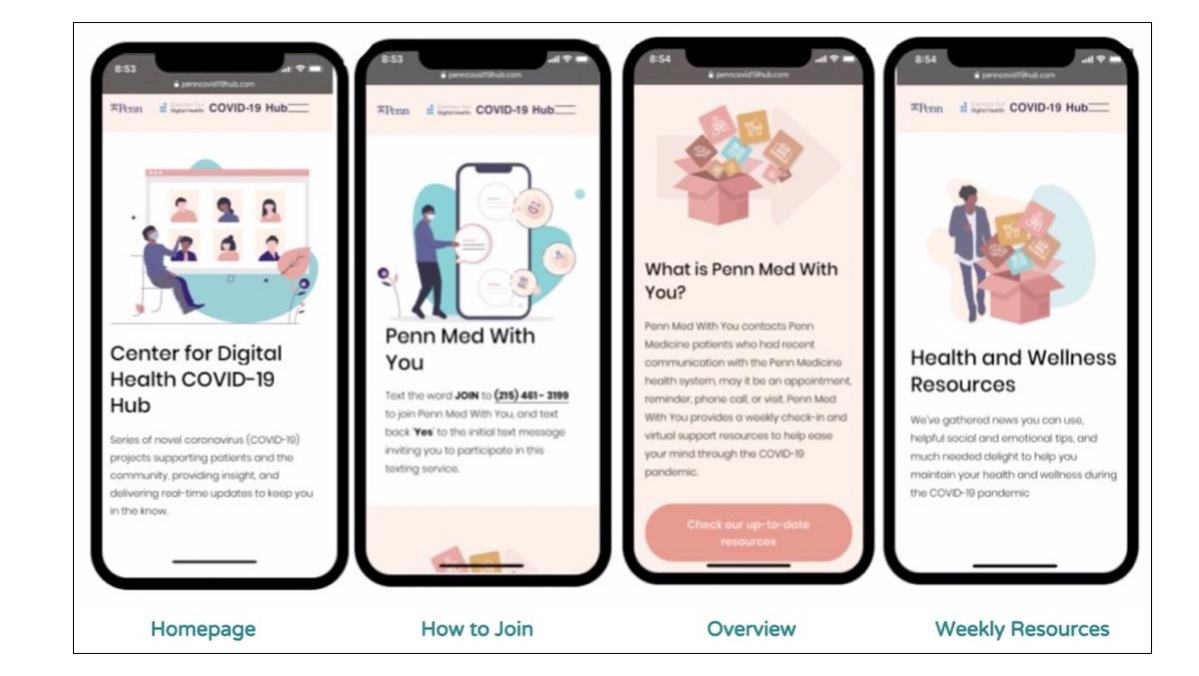
Community-tailored resource hub.

Figures 2-4 display the automated messaging interface and the flow of engagement to connect with individuals. Of note, 90% of the text messages were automated; however, the study team manually reviewed all text messages for quality control and safety audits. For example, after 3 days of low mood ratings, the research team would reach out to the participant to further assess their well-being needs and escalate to a CHW if needed. Messages were sent on weekdays at 10 AM. On weekends, the participants received an out-of-office message. Demographic data were extracted from the participants’ EHR records.

#### Ethical Approval

This study was approved by the University of Pennsylvania Quality Improvement Institutional Review Board. A protocol for identifying risk of emotional and psychological harm was established. We built automated notifications to hover over self-reported well-being scores and free text input. We built automated responses for any messaging that the system could not interpret as an answer and keyword phrases that triggered immediate human intervention (eg, “death,” “kill,” “suicide,” “hang”). This automated messaging directed the participants to seek medical care if needed and directed them to a website for a comprehensive list of other resources. We ultimately established a human mechanism in place to identify the participant, connect with them via telephone, and route them to the appropriate professional.

#### Team

Our team is multidisciplinary, spanning from clinician and nonclinical members. It includes Penn Medicine Center for Digital Health, Center for Healthcare Innovation, department chair and faculty from Emergency Medicine, medical students from the Perelman School of Medicine, data analysts, graphic design students from the University of Pennsylvania, the text messaging platform, and the Individualized Management for Patient-Centered Targets team. The latter is a nationally recognized community health worker program that hires and trains trusted neighborhood residents to become CHWs to carry out culturally appropriate outreach activities, social support, patient advocacy, and health system navigation, with the goal of improving health in underserved populations [18]. The partnership with virtual CHWs provided a robust and well-rounded approach to community health and well-being.

### Statistical Analysis

Descriptive statistics were used to summarize demographic variables. Authors LS and RO categorized the open-ended responses; they reviewed 50 responses to identify common themes and applied a thematic analysis approach [19]. Once categorized, the responses were coded, and a third reviewer adjudicated any discrepancies.

## Results

### User Statistics

The 409 individuals who opted into the “With You” program were asked to self-report their well-being. A total of 286 (70%) individuals responded with a well-being rating. The majority of participants were Black (275/409, 73.5%) and female (264/409, 70.1%) (Table 1). Additional participant demographics and intervention details have been reported previously [15].

**Table 1:** Participant demographics.

Characteristics			Values^a^
Age (years), mean (SD)			46.9 (16.8)
Female, n (%)			264 (70.1)
**Race or ethnicity, n (%)**			
	Black	275 (73.5)	
	White	69 (18)	
	Asian	14 (3)	
	Hispanic Latino	14 (3)	
	Other	16 (4)	
**Emergency department, n (%)**			
	Hospital A	225 (60.2)	
	Hospital B	149 (39.8)	

^a^Of the total study sample (n=383), demographic data were missing for 9 (2.3%) participants.

### Evaluation Outcomes

**Figure 5:**
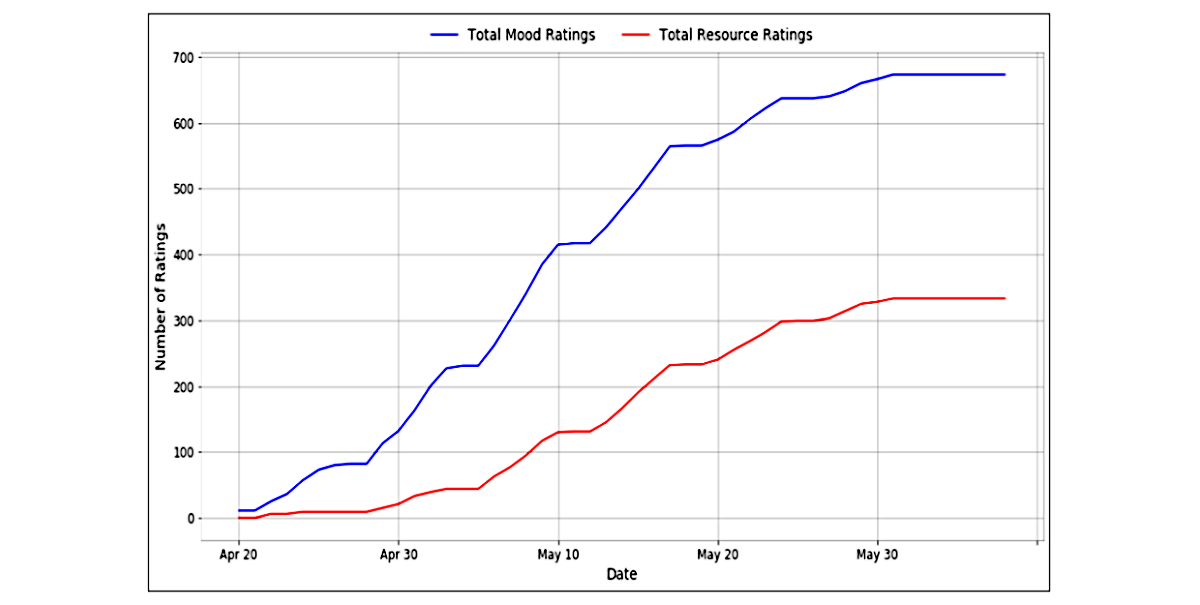
Initial community engagement and mood ratings.

Over the course of 7 weeks, we exchanged 13,917 text messages, elicited 673 self-reported well-being ratings (mean 6.8), and received 333 resource ratings (mean 4) (Figure 5). For those who reported low mood scores, we sent 209 follow-up mood questions. From the follow-up mood questions, only 2% (n=9) of the participants were escalated to CHWs. The website received over 6400 views, and most visits (91.5% [n=5856]) originated from a direct link.

We received 368 open-ended participant responses (Table 2). Most responses were by participants elaborating on their self-reported mood score (132/368, 36%). For example, the system would ask the participants to rate their mood from 0 to 10 (best mood), and a participant followed their score with a message as such:

I am in a good space. Praying for this attack on America to end so that we can get back to a normal existence. On a scale from 1-10 my mood is a 10.

Participant #1, female, aged 58 years

or

My mood is good I say it about 9 thank you for being concerned.

Participant #2, female, aged 56 years

Additionally, after describing one’s mood, most participants (125/368, 34%) would include pleasantries such as the following:

thank you for looking out.

Participant #3, female, aged 35 years

or

I appreciate the text messages, keep them coming!

Participant #4, female, aged 49 years

**Table 2:** Qualitative codes demonstrating themes.

Codes	Operational definition	Frequency, n (%)
Administrative	Question or concern related to text line logistics	55 (15)
Health	Physical and mental health-related question	53 (14)
Mood	Elaborates on mood score response	135 (36.6)
Pleasantries	Responds to automated message	125 (34)

#### Measure of Acceptability

Overall, 77% (164/213) of our participants rated the platform as a service they would highly recommend to a family member or friend. Moreover, 67% (143/213) of unstructured feedback on the platform and content were positive. Through open-ended questions, the participants made the following remarks:

The line is good…Enjoying the information you’re directing to me.

Participant #5, female, aged 55 years

Keep the texting line active. Folks like myself appreciate a caring text and health information/resources. Thank you so much. Be safe….

Participant #5, female, aged 61 years

Thanks, you for crisis info your help is right on time at times I just got to talk to someone thank you once again.

Participant #6, male, aged 29 years

The participants also provided constructive feedback and recommendations through the open-ended questions. This feedback helped guide our platform development. Some examples of unstructured and open feedback are as follows:

Live counselors would be awesome.

Participant #7, female, aged 33 years

More resources for anxiety.

Participant #8, female, aged 40 years

Text at different times of the day to check in.

Participant #9, male, aged 54 years

As we enrolled more participants, we obtained additional feedback on resources and the platform itself as a means to institute continuous and real time quality improvement. For example, a participant requested information on better sleep in the qualitative resource feedback. The following week, we featured recommendations for healthy sleeping habits during COVID-19. The participants also reported feelings of isolation and disconnection; in response, we included content featuring how several neighborhoods were banding together to create outdoor socially distanced events for children.

#### Feasibility and Sustainability

After the pilot study concluded, the research team met twice virtually for approximately 30-45 minutes to elucidate feasibility and sustainability factors. Below are key factors for feasibility and sustainability:

1. Form an interdisciplinary team to frame out the engagement strategy and overarching method of connecting at-risk groups to urgent medical and social needs.
2. Design an approach that learns early and often to inform and adapt growth to needs.
3. Create a central, easy-to-access hub for social resources that currently exist. This includes content for those with low and high self-reported well-being.
4. Build a team and schedule to allow for intermittent but daily monitoring of responses for quality and safety assessment.
5. Recognize that content and resources will need to be tailored to the local community and environments and be open to change.
6. Partner with local champions and resources so as to not reinvent the wheel and lean on the expertise of existing networks.

## Discussion

### Principal Results

This pilot study aimed to determine the acceptability and feasibility of a health system-driven community engagement for health and wellness line. This pilot study engaged discharged ED patients with a visit during the early phase of the pandemic via SMS text messaging to understand their well-being needs, curated a participant-driven repository of community resources, and proactively connected individuals with CHWs to support their well-being and connection to needed resources. In other health systems, CHWs were essential in addressing social determinants of health in vulnerable populations [20]. The pilot demonstrated that health systems are uniquely positioned to serve as hubs and sources of credible information, resources, and connections to community needs. The participants found the SMS text messaging-based line to help with “life needs” and interacted with the text line as if it were a friend, such as asking, “how are you?” saying “stay safe,” and “have a blessed day.” This is noteworthy as these types of responses suggest that health systems are well positioned to remotely address and support community well-being.

The text line also filled an important gap in mental health access. Earlier research found that rates of anxiety and depression are on the rise during the pandemic, and resources are needed [21]. In addition to the emerging mental health concerns, individuals are experiencing an infodemic, ‘‘an overabundance of information—some accurate and some not—that makes it hard for people to find trustworthy sources and reliable guidance when they need it” [22]. Early in the pandemic, the World Health Organization coined a new term and cautioned users about misinformation. Our response was this text line, which focused on providing timely and scientifically accurate information to the participants.

This pilot study demonstrated that digital engagement through simple options such as SMS text messaging provides a means to engage and interact with communities through COVID-19 and other crises. Automated hovering [23], resource sharing, and well-being monitoring act as means to extend the network and expertise of medical centers and can work to support broader community mental health and well-being. As health systems prepare for subsequent waves, additional attention is needed on how to support health care workers (including CHWs) facing the psychological impacts of COVID-19 [24]. As we work to continue to live through and fight COVID-19, health systems are uniquely positioned and can implement rapid, remote, population-level efforts to address and support community health and well-being. The early results from our work provide essential insights toward understanding how health systems can remotely engage and use automated digital technology to explore and react to community needs during times of need. Further research is warranted to explore how individuals navigate social distancing, isolation, health, and well-being throughout the prolonged response to, and recovery from, the pandemic.

### Limitations

This pilot study has limitations. First, it was limited by its enrollment and retention. Overall, 20% of the participants opted into the text messaging line, and 70% were retained. Despite the high rates for a pilot study, it is possible that this contributes to a selection bias. Second, the pilot study only assessed mood and resource ratings from participants who opted in; it did not have comparison or control groups. Our findings only represent community members who are engaged in care at 2 hospitals; these results do not represent those who receive care at other hospitals and most importantly, those who are not engaged in health care at all, which comprises some of the highest need populations. Third, we could not assess how many participants used the resources over the course of the pilot study. This is because the website was publicly available, and nonparticipants might have accessed it. Fourth, we defined our study sample as high-risk due to their ED use during the COVID-19 pandemic. We posit that there are other key variables such as chief complaint and housing stability that would be helpful in further classification. Lastly, we assessed feasibility of a health system implementing a community line; further research can delve into the acceptability of community perspective of the service. Our evaluation methods elicited feedback; however, future research can use frameworks such as the RE-AIM (reach, effectiveness, adoption, implementation, and maintenance) [25] or validated instruments such as Participant Feedback Questionnaire used in other pilot studies [26].

### Comparison With Prior Work

Our pilot study can be compared with prior work, such as the Thought Spot, a randomized controlled trial aimed at optimizing and evaluating a web-based and mobile-based platform designed to improve the ability of students to access mental health and substance use services [27]. Despite the participant demographic differences, both studies employed crowdsourced materials to codesign and cocreate content and focused on participants having an active role in the program to share their knowledge about services and discover wellness tools and resources in their community. Importantly, our pilot study was not a randomized controlled trial and was executed within a short period of 3 months to rapidly respond to mental health and well-being early in the COVID-19 pandemic. Thus, we cannot isolate how seasonal and sociocultural factors in Spring 2020, such as the murder of George Floyd and other African Americans, had an impact on our findings. Interestingly, the topic of CHWs employing digital tools during the COVID-19 is discussed within the context of low-to-middle income countries [28,29]. For example, Feroz, Khoja, and Saleem [29] note how there is some resistance to adopting digital tools among CHWs. Most notably, there is a lack of training on new technologies, weak technical support, and persistent issues of stable internet access.

### Conclusions

This study suggests that health systems are well positioned to support community well-being. It is feasible and acceptable to proactively text health system patients and provide robust, wraparound support during a pandemic. The expanding use of digital technology offers an opportunity to engage community members throughout the stages of the COVID-19 pandemic.

## Abbreviations

CHW: community health worker
ED: emergency department
EHR: electronic health record
HIPAA: Health Insurance Portability and Accountability Act
NPS: net promote score
RE-AIM: reach, effectiveness, adoption, implementation, and maintenance
